# Polycystic ovary syndrome is associated with a higher risk of premalignant and malignant endometrial polyps in premenopausal women: a retrospective study in a tertiary teaching hospital

**DOI:** 10.1186/s12905-023-02269-4

**Published:** 2023-03-24

**Authors:** Ling Lu, Jianbo Luo, Jie Deng, Chaolin Huang, Chanyu Li

**Affiliations:** Department of Gynaecology, The First Affiliated Hospital of Chengdu Medical College, Chengdu, Sichuan Province China

**Keywords:** Polycystic ovary syndrome, Endometrial polyps, Premenopausal women, Malignancy, Hysteroscopic polypectomy

## Abstract

**Background:**

Polycystic ovary syndrome (PCOS) is characterized by anovulation, insufficient progesterone, hyperandrogenism, and insulin resistance. These factors can disrupt the endometrium of PCOS patients and can lead to chronic low-grade inflammation in the endometrium, endometrial hyperplasia, or even endometrial cancer.

**Objective:**

The aim of this study was to investigate the prevalence of premalignant and malignant endometrial polyps in premenopausal women and to further explore whether PCOS is associated with premalignant and malignant changes in endometrial polyps.

**Methods:**

This study was conducted by retrieving the medical data of 4236 premenopausal women who underwent hysteroscopic polypectomies between January 2015 and December 2021. Demographic and clinical data regarding age, height, weight, parity, hormone replacement therapy, oral contraceptives, abnormal uterine bleeding, hypertension, diabetes mellitus, PCOS, number of polyps, and size of polyps were collected, and their associations with premalignant and malignant changes in endometrial polyps were analysed.

**Result:**

Among the endometrial polyps removed by hysteroscopic polypectomy in premenopausal women, the prevalence of premalignant and malignant polyps was 2.15%, which comprised hyperplasia with atypia at 1.13% and endometrial carcinoma at 1.02%. PCOS was associated with a higher risk of premalignant and malignant endometrial polyps in premenopausal women after adjustment for potential confounding factors.

**Conclusion:**

PCOS is associated with a higher risk of premalignant and malignant endometrial polyps in premenopausal women. Therefore, it is important to evaluate the endometrium in PCOS patients with ultrasonography or hysteroscopy, and active management involving hysteroscopic polypectomy should be offered to PCOS patients diagnosed with endometrial polyps regardless of symptoms.

## Introduction

Endometrial polyps are localized overgrowths of endometrial tissue that form finger-like projections from the surface of the endometrium. They consist of stroma, glands, and blood vessels and can be single or multiple [1–3]. According to previous studies, the prevalence of endometrial polyps in women of all age groups ranges from 10 to 40% [4, 5]. Endometrial polyps may be asymptomatic, and the most common symptoms include excessive leukorrhea, abnormal uterine bleeding, and infertility [2, 6]. Most endometrial polyps are benign lesions, but approximately 3–5% of endometrial polyps have been reported as premalignant or malignant [7–9]. However, the factors associated with premalignant and malignant changes in endometrial polyps are not completely understood.

Polycystic ovary syndrome (PCOS) is a common reproductive endocrine disease in women of reproductive age. It has been reported that 5% to 10% of women of reproductive age are diagnosed with PCOS worldwide, and the symptoms of PCOS include amenorrhea, oligomenorrhea, hirsutism, obesity, infertility, and acne [10–13]. PCOS is characterized by anovulation, insufficient progesterone, hyperandrogenism, and insulin resistance. These factors can disrupt the endometrium of PCOS patients and can lead to chronic low-grade inflammation in the endometrium, infertility, endometrial hyperplasia, or even endometrial cancer [13–16]. The risk of endometrial cancer has been shown to be between 2–6 times higher in women with PCOS than in women without PCOS [13]. The pathogenesis of endometrial cancer in PCOS patients is thought to be related to the prolonged stimulation of the endometrium by unopposed oestrogen in the setting of anovulation and prevention of endometrial exfoliation [17]. Thus, it is imperative to determine whether PCOS patients diagnosed with endometrial polyps are at greater risk of developing cancer so that a more judicious indication regarding hysteroscopic polypectomy can be established.

The aim of the current study was to investigate the prevalence of premalignant and malignant endometrial polyps that were removed by hysteroscopy in premenopausal women and to further explore whether PCOS is associated with premalignant and malignant changes in endometrial polyps.

## Materials and methods

### Study design and data collection

This retrospective study was conducted by searching the medical record database for subjects diagnosed with endometrial polyps that were removed by hysteroscopic polypectomy at the First Affiliated Hospital of Chengdu Medical College between January 2015 and December 2021. This hospital is one of the largest tertiary hospitals situated in the megacity of Chengdu in Southwest China. The study complied with the Declaration of Helsinki and was approved by the Ethics Committee of The First Affiliated Hospital of Chengdu Medical College (No. CYFY17143031). Informed consent was obtained from all participants before the study when they visited the outpatient department.

**Fig. 1:**
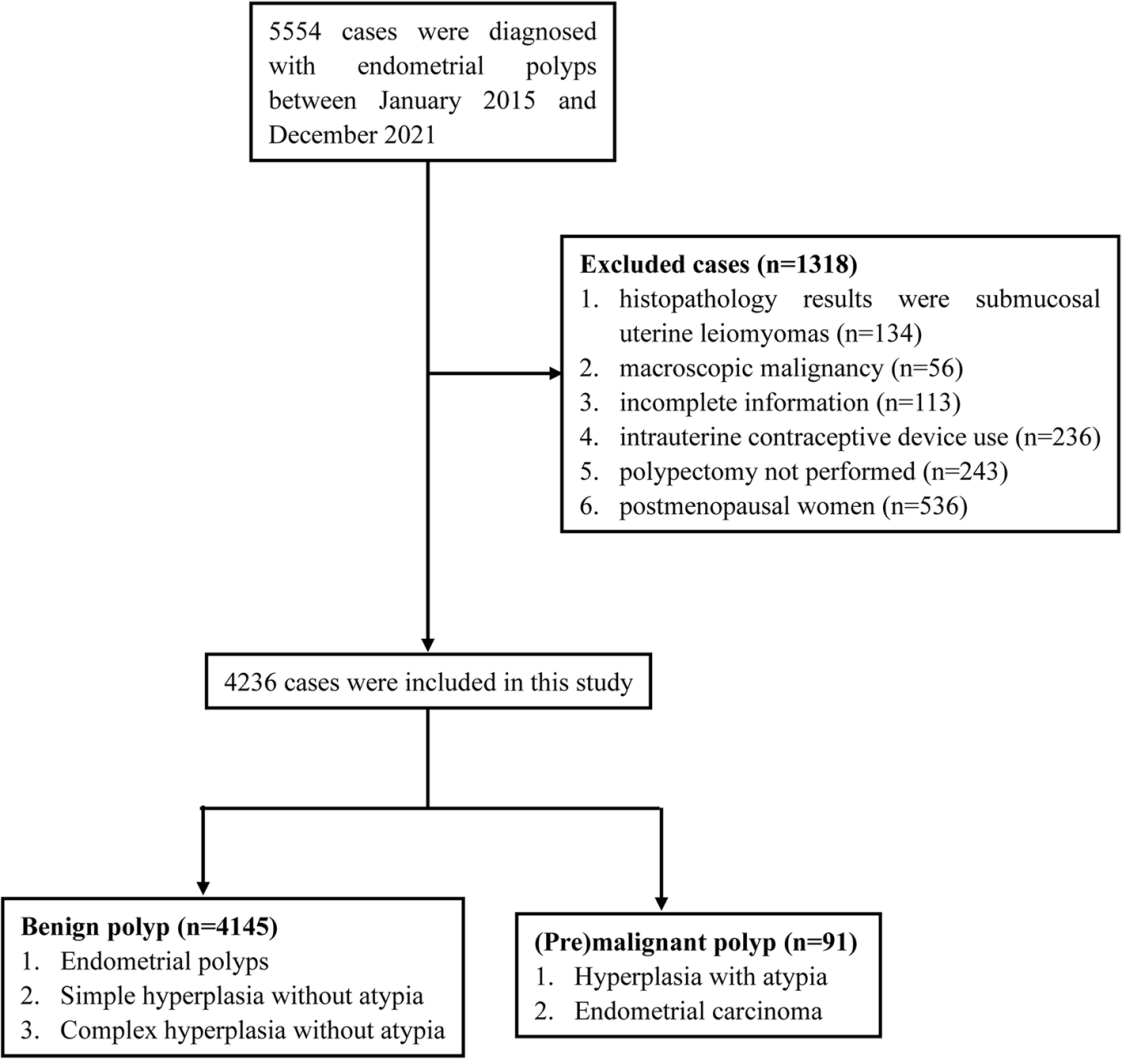
Flowchart of the study design

The study population was selected based on the following inclusion and exclusion criteria. The inclusion criteria were as follows: (1) women who were diagnosed with endometrial polyps either by transvaginal ultrasonography or by hysteroscopy; and (2) premenopausal women. The exclusion criteria were as follows: (1) histopathology results consistent with submucosal uterine leiomyomas; (2) macroscopic malignancy; (3) incomplete information; (4) presence of intrauterine contraceptive device use; and (5) cases where polypectomy was not performed. A total of 4236 women who met the inclusion and exclusion criteria were included in the study. A flowchart of the study design is shown in Fig. 1.

The demographic and clinical characteristics were collected by retrieving information from the medical record database. These included age, height, weight, parity, history of hormone replacement therapy, oral contraceptives, abnormal uterine bleeding, hypertension, diabetes mellitus, PCOS, number of polyps, and size of polyps. The diagnosis of PCOS was based on the Rotterdam criteria that two of the following three criteria should be met: (1) clinical or biochemical hyperandrogenism; (2) ovulatory dysfunction; and (3) polycystic ovaries [18]. The size of the polyps was evaluated by measuring the maximum diameter for a single polyp or the largest polyp in the presence of multiple polyps.

The indications for hysteroscopy were satisfied by women with abnormal uterine bleeding and asymptomatic women in whom the intrauterine lesions were incidental findings during other imaging scans. All the women included in the study had their endometrial polyps removed by hysteroscopic polypectomy. Endometrial assessment was also performed by endometrial aspiration or dilatation and curettage after polypectomy in the same setting. The endometrial polyp specimens and endometrium samples were placed in separate sample bags for histologic examination. In this study, the histologic findings were from the endometrial polyp specimens. The histologic findings of endometrial polyp specimens were defined as endometrial polyp, hyperplasic (simple or complex hyperplasia without atypia), premalignant (hyperplasia with atypia), and malignant (endometrial carcinoma) according to the World Health Organization (WHO) Classification Systems for Endometrial Hyperplasia [19]. Based on the Chinese National Health Commission, obesity was defined as BMI ≥ 28.0 kg/m^2^ in adults [20].

### Statistical analysis

SPSS software (version 16.0, Chicago, IL, USA) was used to analyse statistical significance. Data with normal distributions are presented as the mean ± SD, and nonnormally distributed data are presented as frequencies. The independent-sample *t test* or Mann‒Whitney *U* test was used for continuous variables, and the chi-square test or Fisher’s exact test was used for categorical variables. The association between the premalignant and malignant changes in endometrial polyps and PCOS was determined by multivariate logistic regression analysis after adjustment for potential confounding factors. The results are presented as the odds ratio (OR) and 95% confidence interval (CI). A *p* < 0.05 was considered indicative of statistical significance.

The sample size was calculated using the following formula as described in previous studies: *n* = Z^2^ × P × (1-P)/e^2^, where *n* = the required sample size, Z = 1.96 at a 95% CI, *P* = the prevalence of premalignant and malignant polyps (3%–5%) and e = the margin of error (5%) [21, 22]. PASS (Power Analysis and Sample Size) software was used to evaluate the statistical power and effect size.

## Results

### Histologic findings regarding the endometrial polyps removed from the study population

A total of 4236 women who met the inclusion and exclusion criteria were included in this study. The demographic and clinical characteristics of the study population are shown in Table 1. Histological examination of the removed endometrial polyps showed that the prevalence of the premalignant and malignant polyps was 2.15%. Based on the histologic findings, the specimens were classified into a benign polyp group and a premalignant and malignant polyp group (Table 2). In the benign polyp group, 3057 (72.17%) cases were diagnosed as endometrial polyps, 732 (17.28%) cases were diagnosed as simple hyperplasia without atypia, and 356 (8.4%) cases were diagnosed as complex hyperplasia without atypia. In the premalignant and malignant polyp group, 48 (1.13%) cases were diagnosed as hyperplasia with atypia, and 43 (1.02%) cases were diagnosed as endometrial carcinoma (Table 2).

**Table 1:** The demographic and clinical characteristics of the study population (*n* = 4236)

Parameters	Value
**Age (year)**	42 ± 7.5
**BMI (kg/m**^**2**^**)**	26.53 ± 4.3
**Menopausal status**	
premenopausal	4236
postmenopausal	0
**Gravidity**	
0	534 (12.61%)
≥ 1	3702 (87.39%)
**Parity**	
0	704 (16.62%)
≥ 1	3532 (83.38%)
**Abnormal uterine bleeding**	
Yes	1045 (24.67%)
No	3191 (75.33%)
**Hypertension**	
Yes	516 (12.18%)
No	3720 (87.82%)
**Diabetes mellitus**	
Yes	749 (17.68%)
No	3487 (82.32%)
**PCOS**	
Yes	337 (7.96%)
No	3899 (92.04)
**Hormone replacement therapy**	
Yes	168 (3.97%)
No	4068 (96.03%)
**Oral contraceptives**	
Yes	501 (11.83%)
No	3735 (88.17%)
**Polyp number**	
1	3012 (71.1%)
≥ 2	1224 (28.9%)
**Polyp size (cm)**	
< 2	2994 (70.68%)
≥ 2	1242 (29.32%)

**Table 2:** Histologic findings regarding the endometrial polyp removed from the study population (*n* = 4236)

Histology category	Frequency (%)
**Benign polyp**	4145 (97.85)
Endometrial polyps	3057 (72.17)
Simple hyperplasia without atypia	732 (17.28)
Complex hyperplasia without atypia	356 (8.4)
**(Pre)malignant polyp**	91 (2.15)
Hyperplasia with atypia	48 (1.13)
Endometrial carcinoma	43 (1.02)

### Univariate analysis of risk factors for premalignant and malignant endometrial polyps

Univariate analysis was performed to identify risk factors for premalignant and malignant endometrial polyps. The results showed that age (≥ 40 years), obesity, nulliparity, diabetes mellitus, PCOS, and polyp number were significantly associated with a higher risk of premalignant and malignant polyps (*p* < 0.05) (Table 3). The ORs (95% CIs) for age (≥ 40 years), obesity, nulliparity, diabetes mellitus, PCOS, and polyp number were 1.74 (1.12–2.71), 1.64 (1.07–2.53), 2.8 (1.81–4.35), 3.3 (2.16–5.06), 2.96 (1.74–5.02), and 1.71 (1.12–2.61), respectively. However, no statistically significant associations were observed between the premalignant and malignant polyps and other variables, including abnormal uterine bleeding, gravidity, hypertension, history of hormone replacement therapy, oral contraceptives, and the size of polyps (*p* > 0.05) (Table 3).

**Table 3:** Univariate analysis of risk factors for premalignant and malignant endometrial polyps (*n* = 4236)

Variable	(Pre)Malignant polyps *n* = 91	Benign polyps *n* = 4145	OR	95% CI	*p-value*
**Age**			1.74	1.12–2.71	0.017
≥ 40	61	2231			
< 40	30	1914			
**Obesity (BMI ≥ 28.0 kg/m**^**2**^**)**			1.64	1.07–2.53	0.03
Yes	58	2143			
No	33	2002			
**Gravidity**					
0	17	517	1.61	0.94–2.75	0.108
≥ 1	74	3628			
**Parity**			2.8	1.81–4.35	<0.0001
0	32	672			
≥ 1	59	3473			
**Abnormal uterine bleeding**			1.37	0.87–2.15	0.172
Yes	28	1017			
No	63	3128			
**Hypertension**			1.319	0.74–2.35	0.434
Yes	14	502			
No	77	3643			
**Diabetes mellitus**			3.30	2.16–5.06	<0.0001
Yes	37	712			
No	54	3433			
**PCOS**			2.96	1.74–5.02	<0.0001
Yes	18	319			
No	73	3826			
**Hormone replacement therapy**			2.06	0.94–4.53	0.117
Yes	7	161			
No	84	3984			
**Oral contraceptives**			1.49	0.85–2.61	0.220
Yes	15	486			
No	76	3659			
**Polyp number**			1.71	1.12–2.61	0.012
2 ≥	37	1187			
1	54	2958			
**Polyp size (cm)**			0.66	0.43–1.01	0.069
2 ≤	56	2938			
> 2	35	1207			

### Multivariate analysis of risk factors for premalignant and malignant endometrial polyps

The risk factors that were identified by univariate analysis were further evaluated by a multivariate logistic regression analysis model. The results showed that only PCOS was significantly associated with a higher risk of premalignant and malignant endometrial polyps after adjustment for confounding factors, including age, obesity, parity, diabetes mellitus, and polyp number; the OR (95% CI) was 2.75 (1.02–3.45) (Table 4). This finding suggested that PCOS was an independent risk factor for premalignant and malignant endometrial polyps. The PCOS factor was also used to build a risk prediction model for premalignant and malignant polyps. An ROC curve was constructed, and the AUC was 0.746 with a sensitivity of 72.3% and a specificity of 81.4%.

**Table 4:** Multivariate analysis of risk factors for premalignant and malignant endometrial polyps

Variables	OR	95% CI	*p-value*
Age	1.32	0.95—2.07	0.113
Obesity	2.45	1.02—3.41	0.185
Parity	3.23	2.15—4.27	0.089
Diabetes mellitus	4.22	3.02—5.87	0.221
Polyp number	1.23	0.35—2.17	0.217
PCOS	2.75	1.02—3.45	< 0.001

Adjustment for age, obesity, parity, diabetes mellitus, and polyp number

### The association between PCOS and different histological types of endometrial polyps

**Fig. 2:**
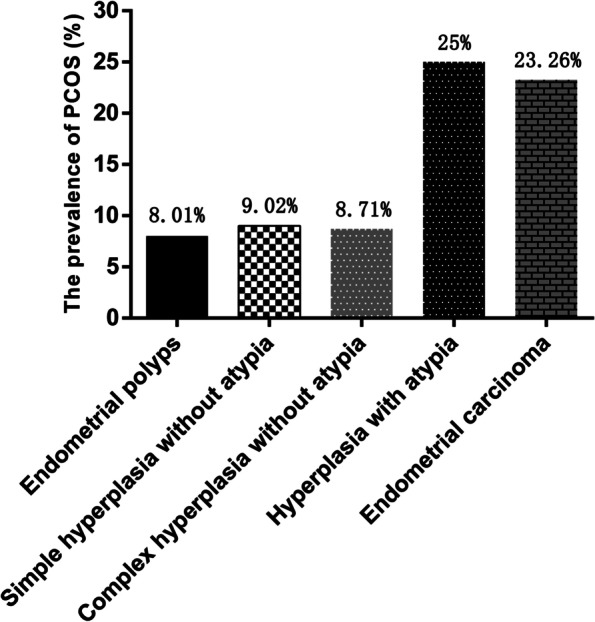
The prevalence of PCOS in different histological types of endometrial polyps

The prevalence of PCOS was evaluated in different histological types of endometrial polyps, and the results showed that the prevalence of PCOS was 8.01% in endometrial polyps, 9.02% in simple hyperplasia without atypia, 8.71% in complex hyperplasia without atypia, 25% in hyperplasia with atypia, and 23.26% in endometrial carcinoma (Fig. 2). The association between PCOS and different histological types of endometrial polyps was further evaluated by logistic regression analysis (Table 5). The results showed that PCOS was significantly associated with a higher risk of hyperplasia with atypia and endometrial carcinoma; the ORs (95% CIs) were 3.96 (2.04–7.69) and 3.58 (1.75–7.34), respectively (Table 5). However, no associations were observed between PCOS and endometrial polyps, simple hyperplasia without atypia, and complex hyperplasia without atypia (*p* > 0.05).

**Table 5:** The association between PCOS and different histological types of endometrial polyps (*n* = 4236)

Histology category	PCOS (*n* = 337)	Non-PCOS (*n* = 3899)	OR	95%CI	*p-value*
Endometrial polyps (*n* = 3057)	245	2812	1.03	0.80–1.32	0.87
Simple hyperplasia without atypia (*n* = 732)	66	666	1.18	0.89–1.57	0.275
Complex hyperplasia without atypia (*n* = 356)	31	325	1.11	0.757–1.64	0.656
Hyperplasia with atypia (*n* = 48)	12	36	3.96	2.04–7.69	< 0.0001
Endometrial carcinoma (*n* = 43)	10	33	3.58	1.75–7.34	0.0006

## Discussion

Our study revealed that the prevalence of premalignant and malignant endometrial polyps was 2.15%, comprising rates of 1.13% for hyperplasia with atypia and 1.02% for endometrial carcinoma, among the polyps removed by hysteroscopic polypectomy in premenopausal women (Table 2). Moreover, PCOS was found to be associated with a higher risk of premalignant and malignant endometrial polyps in premenopausal women after adjustment for potential confounding factors (Table 4). These findings may provide guidance for clinical practice in the management of endometrial polyps among premenopausal women with PCOS.

A meta-analysis study that included 35,345 premenopausal and postmenopausal women showed that the prevalence of premalignant and malignant endometrial polyps was 2.73% [8]. Another meta-analysis study involved recruitment of 21,057 patients and reported that 3.4% of patients presented with premalignant and malignant endometrial polyps [9]. However, our study revealed that the prevalence of premalignant endometrial polyps was 1.13%, and the prevalence of malignant endometrial polyps was 1.02% (Table 2). The discrepancies could be attributed to differences in the characteristics of the study populations; these other studies included premenopausal women as well as postmenopausal women. However, only premenopausal women were included in our study, which may explain the lower prevalence in our population compared to that in the populations of these other studies when considering menopause as a risk factor for malignant changes in endometrial polyps [4]. Definition of the standards of obesity may also be an important factor that contributes to the difference in the prevalence of premalignant and malignant polyps between our population and others. We used the Chinese criteria BMI ≥ 28.0 kg/m^2^ to define obesity, while the other studies used the World Health Organization recommended criteria BMI ≥ 30.0 kg/m^2^ to define obesity [20]. Another explanation for the discrepancies might be attributed to the different methods used for the removal of polyps. Some studies performed the removal of polyps by uterine curettage, which usually fails to extract the whole polyp and obtains only a mixed specimen of polyps and endometrial mucosae. This may result in inconsistency of histological diagnosis. Our study involved performing the removal of polyps by hysteroscopic polypectomy, which is a more reliable technique for removing the entire polyp and allowing a more complete histological examination.

In the univariate analysis, we found that age (≥ 40 years), obesity, nulliparity, diabetes mellitus, PCOS, and polyp number were significantly associated with the risk of premalignant and malignant polyps in premenopausal women. However, analysed by a multivariate logistic regression model, only PCOS was found to be significantly associated with the risk of premalignant and malignant polyps when potential confounding factors were controlled (Table 4). The prevalence of hyperplasia with atypia and endometrial carcinoma was 3.96 times and 3.58 times greater in women with PCOS than in those without PCOS (Table 5). This may be explained by the operation of a mechanism that involves endocrinologic and metabolic disorders in PCOS, specifically chronic anovulation, hyperandrogenism, and insulin resistance [17]. Chronic anovulation results in endometrial proliferation by long-term exposure to oestrogen without the opposing action of progesterone [23, 24]. Androgens can convert to oestrogens and indirectly stimulate endometrial proliferation [25]. Insulin resistance is accompanied by hyperinsulinism, which increases the levels of free androgen in the plasma by reducing the production of sex hormone-binding globulin, and high levels of androgen and insulin in the plasma can affect endometrial cell differentiation [26, 27].

In the present study, the prevalence of premalignant and malignant polyps was not associated with the presence of abnormal uterine bleeding. However, other studies have reported that abnormal uterine bleeding is associated with an increased risk of malignant polyps in postmenopausal women [28]. The discrepancy may be attributed to our study including only premenopausal women. It may also be explained by the fact that some small premalignant and malignant lesions existed on the surface or inside the polyps that were detected only by the pathological examinations and did not cause abnormal uterine bleeding. Therefore, when women are diagnosed with endometrial polyps, we propose that active management involving hysteroscopic polypectomy should be offered to premenopausal women with PCOS regardless of symptoms and to postmenopausal women with symptoms. Moreover, although screening for premalignant and malignant polyps with hysteroscopy is not recommended when considering the invasive nature of the procedure and the economic costs, we recommend routine pelvic ultrasonography for premenopausal women as well as for postmenopausal women regardless of symptoms. Evidence-based management of incidental ultrasound findings such as endometrial polyps has gained importance for modern gynaecologists. Therefore, it will be of great value to understand the significance of both symptomatic and asymptomatic endometrial polyps and their proposed management.

Our study has many strengths. First, this study included a large sample size, and polyp removal was performed by a standardized procedure. Second, potential confounding factors were controlled in our evaluation of the association between PCOS and the premalignant and malignant endometrial polyps. Third, this study focused on premenopausal women because a considerable proportion of endometrial polyps are asymptomatic and are found incidentally in premenopausal women. Nevertheless, this study also has limitations. First, this study was conducted by a retrospective review of patient data and is subject to potential selection bias. Second, although a variety of potential confounding factors were controlled when we evaluated the association between PCOS and the premalignant and malignant endometrial polyps in premenopausal women, we cannot rule out the effect of any residual confounding factors on the findings. Third, the number of PCOS cases may have been underestimated in this study because we did not routinely screen PCOS in all outpatients. Finally, since this is a single-centre study, further multicentre studies are needed to confirm our findings.

## Conclusions

PCOS is associated with a higher risk of premalignant and malignant endometrial polyps in premenopausal women. Therefore, it is necessary to evaluate the endometrium in PCOS patients with ultrasonography or hysteroscopy, and active management involving hysteroscopic polypectomy should be offered to PCOS patients diagnosed with endometrial polyps regardless of symptoms.

## Data Availability

Datasets are held by The First Affiliated Hospital of Chengdu Medical College and stored on the website: https://pan.baidu.com/s/1T62SehYZHjhBP2nG0NIYow. Any researchers can access to the datasets by contacting the corresponding author and providing recommendations concerning non-commercial use and privacy protection from their institutions.
